# Value of Peak Expiratory Flow Rate in Evaluating Cough Ability in Patients Undergoing Lung Surgery

**DOI:** 10.1155/2021/5888783

**Published:** 2021-12-15

**Authors:** Gui-Xian Liu, Jian-Hua Su, Xin Wang, Jin-Tao He

**Affiliations:** ^1^Southwest Medical University, Luzhou 646000, Sichuan, China; ^2^Department of Rehabilitation, West China Hospital, Sichuan University, Chengdu 610000, Sichuan, China; ^3^Department of Thoracic Surgery, Sichuan Cancer Hospital, Chengdu 610041, Sichuan, China

## Abstract

**Introduction:**

Postoperative ineffective cough is easy to occur after thoracic surgery, and it is also a risk factor for postoperative pulmonary complications (PPCs).

**Objectives:**

To explore the value of peak expiratory flow rate (PEF) in evaluating cough ability in patients undergoing lung surgery and evaluate the effectiveness of chest wall compression during the expiratory phase by PEF.

**Methods:**

From September 2020 to May 2021, the researchers collected the data of patients who underwent lung surgery. Eventually, 153 patients who met the criteria were included, 102 cases were included in the effective cough group and 51 cases were included in the ineffective cough group. The receiver working curve (ROC curve) was used to analyze whether PEF could evaluate cough ability. At the same time, the researchers collected the pulmonary function data of the first 30 patients of the ineffective cough group while compressing the chest wall during the expiratory phase to evaluate the effectiveness of chest wall compression.

**Results:**

The area under the curve (AUC) of postoperative PEF to evaluate the postoperative cough ability was 0.955 (95% CI: 0.927–0.983, *P* < 0.001). The values of PEF (127.17 ± 34.72 L/min vs. 100.70 ± 29.98 L/min, *P* < 0.001, 95% CI: 18.34–34.59) and FEV_1_ (0.72 (0.68–0.97) L vs. 0.64 (0.56–0.82) L, *P* < 0.001) measured while compressing the chest wall were higher than those without compression.

**Conclusions:**

PEF can be used as a quantitative indicator of cough ability. Chest wall compression could improve cough ability for patients who have ineffective cough.

## 1. Introduction

Thoracic surgery often causes a certain degree of damage to the chest wall and lung parenchyma, which might lead to decreased cough ability. The secretions cannot be effectively eliminated, which may easily lead to pulmonary infections [1]. Effective cough could prevent postoperative pulmonary infection and atelectasis by clearing secretions and keeping the airway unobstructed [2]. At present, only a few indicators were used in evaluating the cough ability after surgery, such as measuring the volume of sputum excreted and observing the occurrence of PPCs. However, there are great differences in the volume of sputum excreted among different patients. Meanwhile, the occurrence of PPCs is affected by many factors. Therefore, an objective quantitative index is needed to evaluate the cough ability.

PEF can reflect the strength of respiratory muscles and the patency of airway [3, 4], and it has been used to predict PPCs after lung surgery [5]. However, there was no relevant research reported on whether PEF could be used as the quantitative index in evaluating postoperative cough ability. This study aims to explore postoperative PEF's role in evaluating cough ability for the patients undergoing lung surgery; meanwhile, we also explore the effectiveness of chest wall compression during the expiratory phase by evaluating postoperative PEF.

## 2. Materials and Methods

### 2.1. Sample Quantity Estimation

In the first part, it was a diagnostic test. The hypothesis of this study was that PEF can distinguish between effective cough and ineffective cough. According to the preexperiment results, the incidence of ineffective cough on the first day after surgery was about 20%, and the sensitivity and specificity of PEF were 85% and 99%. *α* = 0.05 was set, power was 0.9, and then the minimum sample amount was calculated by using PASS 15.0. The values based on sensitivity and specificity were 85 and 8, respectively. 68 patients in the effective cough group and 17 patients in the ineffective cough group needed to be included at least. In order to increase the credibility of the research results, this study would prolong the enrollment time of the ineffective cough group to ensure that the ratio of effective cough to ineffective cough was 2 : 1. In the second part, it was a self-paired sample. *α* = 0.05 was set, power was 0.9, and then the minimum sample amount was calculated by using PASS 15.0. The values of the nonparametric test and the self-paired *T*-test were 10 and 23.

### 2.2. Inclusion and Exclusion Criteria

  Inclusion criteria: (1) the preoperative diagnosis was pulmonary carcinoma or suspected pulmonary carcinoma; (2) treated by surgery (including thoracotomy or thoracoscopic lobectomy and sublobectomy); (3) had complete clinical data; (4) the patient was willing to participate in the study and sign the informed consent form
  Exclusion criteria: (1) patients with pulmonary infection or severe cardio-cerebral-renal comorbidities before operation; (2) patients with severe postoperative complications such as tension pneumothorax and pulmonary embolism; (3) patients with unstable vital signs on the first day after operation, such as hypoxemia and tachyarrhythmia

### 2.3. Research Object and Grouping Method

**Figure 1:**
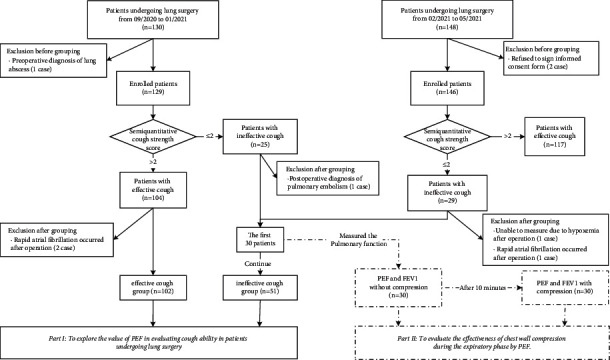
The flowchart for patients' inclusion and groups.

There were two prospective studies. In the first part, from September 2020 to January 2021, 130 consecutive patients of the Department of Thoracic Surgery of Sichuan Cancer Hospital were enrolled into the study. Based on the semiquantitative cough strength score (Table 1) and the international standards, ineffective cough was defined if the cough strength score was not more than 2 [6, 7]. And 126 patients who met the inclusion and exclusion criteria were divided into the effective cough group (*n* = 102) and the ineffective cough group (*n* = 24). From February 2021 to May 2021, 148 consecutive patients of the institution were enrolled, and 27 patients who met the inclusion and exclusion criteria and semiquantitative cough strength score ≤2 were added to the ineffective cough group. Eventually, 153 patients were included in this study, including 102 patients with effective cough and 51 patients with ineffective cough. The pulmonary function of the patients was measured from 17:00 to 19:00 on the day before operation and from 17:00 to 19:00 on the first day after operation. It was measured repeatedly for three times after the correct demonstration every day, and the highest value was recorded. In the second part, for the first 30 patients in the ineffective cough group, the pulmonary function was measured again while compressing the chest wall during the expiratory phase after a ten-minute rest, and the measuring method was the same as before (Figure 1). All participants gave written informed consent. And the program was approved by the ethics committee of Sichuan Cancer Hospital.

### 2.4. Statistics

SPSS 26.0 statistical software was used to analyze the data. The data of normal distribution were described by $\bar{X}\pm S$, and the data of nonnormal distribution were described by *M* (*P*_25_, *P*_75_). For the convenience of comparison, the data in Table 2 were described in *M* (*P*_25_, *P*_75_) because most of the data were of nonnormal distribution. *T*-test and rank-sum test were used for comparison of the continuous data between groups, and chi-square test was used for comparison of classified data between groups. The receiver working curve (ROC curve) was used to analyze whether PEF could evaluate coughing ability. A self-paired *t*-test or signed rank-sum test was used to evaluate the auxiliary effect of chest wall compression on cough. The two-sided test was used in all the statistical tests, and *P* < 0.05 was considered to indicate statistical significance.

## 3. Results

**Figure 2:**
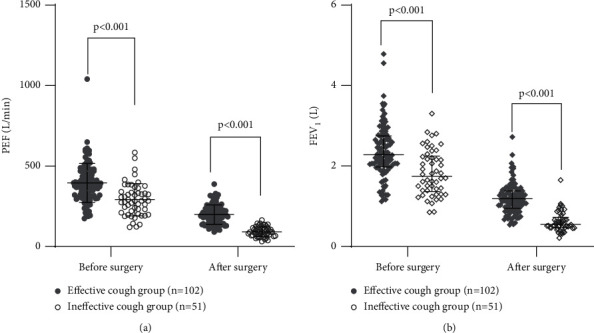
Scatter plot of lung function of two groups of patients before and after surgery.

In the first part, from September 2020 to January 2021, 126 consecutive patients of the Department of Thoracic Surgery of Sichuan Cancer Hospital were included in the study, and they were divided into the effective cough group (*n* = 102) and the ineffective cough group (*n* = 24). From February 2021 to May 2021, 27 patients who met the inclusion and exclusion criteria and semiquantitative cough strength score ≤2 were added to the ineffective cough group. Eventually, 153 patients were included in this study, including 102 patients with effective cough and 51 patients with ineffective cough. Baseline patient characteristics are presented in Table 2. There was no significant difference in gender, BMI, or smoking between the two groups (*P* > 0.05). Compared with the effective cough group, the ineffective cough group had older age, the higher proportion of preoperative respiratory comorbidities, and the higher proportion of pulmonary lobectomy (*P* < 0.05). The mean values of PEF and the median of FEV_1_ before operation and those on the first day after operation in the ineffective cough group were significantly lower than those in the effective cough group (*P* < 0.05) (Figure 2).

**Figure 3:**
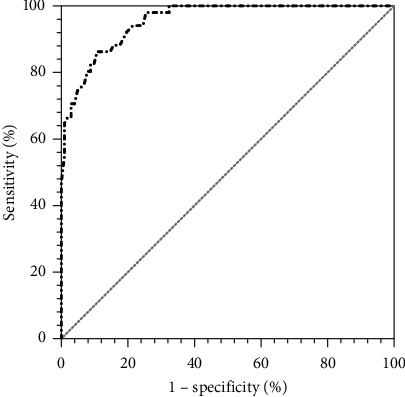
The ROC curve of PEF for evaluating cough ability; the AUC of the ROC curve was 0.955 (95% CI: 0.927–0.983, *P* < 0.001).

The AUC of the ROC curve was 0.955 (95% CI: 0.927–0.983, *P* < 0.001) (Figure 3). When the Youden index (sensitivity + specificity − 1) reached the maximum, the best cutoff value of PEF was 128.5 L/min (sensitivity: 89.2% and specificity: 86.3%).

**Figure 4:**
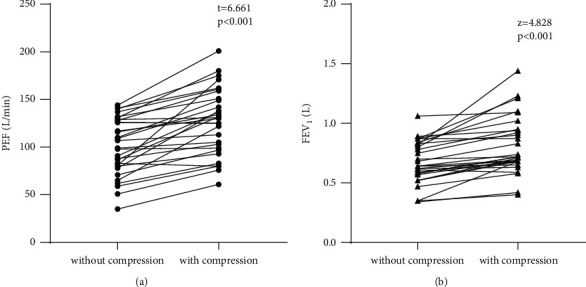
The effect of chest wall compression during the expiratory phase on PEF and FEV_1_.

In the second part, the value of PEF measured during compressing the chest wall (127.17 ± 34.72 L/min) was significantly higher than that without compression (100.70 ± 29.98 L/min), and the mean of difference value was 26.47 ± 21.76 L/min (*P* < 0.001, 95% CI: 18.34–34.59) (Figure 4(a)). The median of FEV_1_ measured during compressing the chest wall was 0.72 (0.68–0.97) L, while that without compression was 0.64 (0.56–0.82) L; the difference was statistically significant (*p* < 0.001) (Figure 4(b)).

## 4. Discussion

The process of measuring PEF has the same mechanism as cough; that is, the rapid contraction of the expiratory muscle leads to the decrease of chest volume and the rapid exhalation of high-pressure gas [8]. Zhou et al.'s study suggested that PEF could be used as a predictor of PPCs because PEF was thought to reflect expiratory muscle strength and the efficacy of cough [5]. In this study, we confirmed that the PEF could be a quantitative measurement of cough ability. PEF is an effective way to observe the cough ability of patients after operation, and it is a convenient indicator for dynamic observation and recording.

If the ineffective cough occurred after operation, timely detection and effective measures will also help to prevent the occurrence of PPCs. Commonly used clinical auxiliary cough and expectoration methods include airway atomization, knocking on the back, vibration expectoration, pressing stimulation, and compressing the chest wall [9–12]. The ineffective cough caused by decreased muscle strength could be solved by compressing the chest wall. Compressing the chest wall in the expiratory phase could directly increase the expiratory power and effectively assist expectoration. In this study, similar results were observed in patients with ineffective cough. Compressing the chest wall does not need the assistance of drugs and appliances, and there are no special complications and adverse reactions. It is a safe, economical, and effective auxiliary means of cough in the clinic.

## Figures and Tables

**Table 1:** Semiquantitative cough strength score.

Score	The condition of coughing
0	No cough on command
1	Visible movement but no audible cough
2	Weakly (barely) audible cough
3	Clearly audible cough
4	Stronger cough
5	Multiple sequential strong coughs

**Table 2:** Baseline patient characteristics of the two groups.

	General population	Group		Statistic	*P*
		Ineffective cough (*n* = 51)	Effective cough (*n* = 102)		
Age (*M* (*P*_25_, *P*_75_), year)	56 (48, 67)	64 (55, 69)	52.5 (47, 64)	−3.313	0.001
BMI (*M* (*P*_25_, *P*_75_), (kg/m^2^))	22.77 (20.82, 25.15)	22.86 (20.81, 26.22)	22.71 (20.95, 24.91)	−0.161	0.872
Sex					
Male	61 (39.9%)	16 (31.4%)	45 (44.1%)	2.304	0.129
Female	92 (60.1%)	35 (68.6%)	57 (55.9%)		
Smoking					
No	104 (68.0%)	35 (68.6%)	69 (67.6%)	0.015	0.902
Yes	49 (32.0%)	16 (31.4%)	33 (32.4%)		
Respiratory complication					
No	122 (79.7%)	31 (60.8%)	91 (89.2%)	17.011	<0.001
Yes	31 (20.3%)	20 (9.2%)	11 (10.8%)		
Mode of operation					
Lobectomy	112 (73.2%)	45 (88.2%)	67 (65.7%)	8.813	0.003
Sublobectomy	41 (26.8%)	6 (11.8%)	35 (34.3%)		

## Data Availability

The data that support the findings of this study are available from the corresponding author upon reasonable request.
